# Genome Investigation of Urinary *Gardnerella* Strains and Their Relationship to Isolates of the Vaginal Microbiota

**DOI:** 10.1128/mSphere.00154-21

**Published:** 2021-05-12

**Authors:** Catherine Putonti, Krystal Thomas-White, Elias Crum, Evann E. Hilt, Travis K. Price, Alan J. Wolfe

**Affiliations:** a Department of Microbiology and Immunology, Stritch School of Medicine, Loyola University Chicago, Maywood, Illinois, USA; b Bioinformatics Program, Loyola University Chicago, Chicago, Illinois, USA; c Department of Biology, Loyola University Chicago, Chicago, Illinois, USA; d Department of Microbiology and Immunology, Stanford University School of Medicine, Stanford, California, USA; University of Michigan-Ann Arbor

**Keywords:** *Gardnerella*, urinary microbiome, phylogenomics, lower urinary tract symptoms

## Abstract

*Gardnerella* is a frequent member of the urogenital microbiota. Given the association between Gardnerella vaginalis and bacterial vaginosis (BV), significant efforts have been focused on characterizing this species in the vaginal microbiota. However, *Gardnerella* also is a frequent member of the urinary microbiota. In an effort to characterize the bacterial species of the urinary microbiota, we present here 10 genomes of urinary *Gardnerella* isolates from women with and without lower urinary tract symptoms. These genomes complement those of 22 urinary *Gardnerella* strains previously isolated and sequenced by our team. We included these genomes in a comparative genome analysis of all publicly available *Gardnerella* genomes, which include 33 urinary isolates, 78 vaginal isolates, and 2 other isolates. While once this genus was thought to consist of a single species, recent comparative genome analyses have revealed 3 new species and an additional 9 groups within *Gardnerella*. Based upon our analysis, we suggest a new group for the species. We also find that distinction between these *Gardnerella* species/groups is possible only when considering the core or whole-genome sequence, as neither the sialidase nor vaginolysin genes are sufficient for distinguishing between species/groups despite their clinical importance. In contrast to the vaginal microbiota, we found that only five *Gardnerella* species/groups have been detected within the lower urinary tract. Although we found no association between a particular *Gardnerella* species/group(s) and urinary symptoms, further sequencing of urinary *Gardnerella* isolates is needed for both comprehensive taxonomic characterization and etiological classification of *Gardnerella* in the urinary tract.

**IMPORTANCE** Prior research into the bacterium Gardnerella vaginalis has largely focused on its association with bacterial vaginosis (BV). However, *G. vaginalis* is also frequently found within the urinary microbiota of women with and without lower urinary tract symptoms as well as individuals with chronic kidney disease, interstitial cystitis, and BV. This prompted our investigation into *Gardnerella* from the urinary microbiota and all publicly available *Gardnerella* genomes from the urogenital tract. Our work suggests that while some *Gardnerella* species can survive in both the urinary tract and vagina, others likely cannot. This study provides the foundation for future studies of *Gardnerella* within the urinary tract and its possible contribution to lower urinary tract symptoms.

## Abstract (precis)

Prior research into the bacterium Gardnerella vaginalis has largely focused on its association with bacterial vaginosis (BV). However, *G. vaginalis* is also frequently found within the urinary microbiota of women with and without lower urinary tract symptoms as well as individuals with chronic kidney disease, interstitial cystitis, and BV.

## INTRODUCTION

Bacterial vaginosis (BV) is defined as a dysbiosis of the vaginal microbiota that leads to irritation of the vaginal tract and is associated with increased risk of preterm birth and sexually transmitted infections (1, 2). While the cause of BV is unknown, many suspect Gardnerella vaginalis to be either the cause or a biomarker of this dysbiosis. However, *G. vaginalis* also is frequently found within the vaginal and urinary microbiotas of women without BV (3, 4). Therefore, research into *G. vaginalis* pathogenicity has been inconclusive (5).

Whole-genome sequencing of *G. vaginalis* isolates from the urogenital microbiota has incited reevaluation of the genus and species. *Gardnerella* genomes have been separated into variants (6), genotypes (7, 8), genovars (9), or ecotypes (10), but all contain 16S rRNA gene sequences with >98% similarity. The use of other marker genes (e.g., *cpn60*) has led to phylogenies distinct from that determined by the use of the 16S rRNA gene (11, 12). The most recent whole-genome analysis identified at least 13 separate species/groups within the genus, including the description of three new species in addition to *G. vaginalis*: G. leopoldii, G. piotii, and G. swidsinskii (13). These 13 species/groups, which fall into eight major clades, can be differentiated by both their allelic variation within the core genes and the gene content of their accessory genomes (14).

Given the definition of these new species/groups, a new hypothesis is posed: only some *Gardnerella* species/groups are associated with BV, while others can be considered part of the normal vaginal microbiota (4, 12, 15). Prior studies have found that multiple *Gardnerella* species/groups are frequently present within an individual’s vaginal microbiota (12), including the vaginal microbiotas of women with BV (16–18). Furthermore, recent work suggests that BV may be a polymicrobial infection that can include multiple different *Gardnerella* species/groups (19–23).

While the majority of research into *Gardnerella* has focused on its prevalence in the vaginal microbiota, it is also frequently present within the bladders of women with and without lower urinary tract symptoms (24–31). Moreover, *Gardnerella* has been regularly detected within midstream voided urine samples of individuals with chronic kidney disease (32), interstitial cystitis (33, 34), and BV (35). Prior research also has suggested associations between BV and urinary tract infections (UTI) (see the work of Morrill et al. (23) and references therein), and it has been shown that uropathogens can associate with and enhance *G. vaginalis* biofilms (36, 37). Regarding the bladder, periurethral, and urethral microbiotas, *Gardnerella* is more common within younger, premenopausal women (30, 38). As all of the aforementioned investigations relied on 16S rRNA sequencing to detect *Gardnerella* within the microbiome, it is not possible to determine the *Gardnerella* species/group(s) present. Thus, associations between urinary tract symptoms and the newly defined species/groups is unknown.

In an effort to characterize the bacterial species of the urinary microbiota, we previously isolated and sequenced 22 *Gardnerella* strains (28, 39). These strains supplement publicly available genomes, most of which belong to isolates from vaginal samples. Here, we present an additional 10 genomes of urinary *Gardnerella* isolates. With these new genomes available, we conducted a comparative genome analysis of all publicly available *Gardnerella* genomes. In total, 113 genomes were examined. Our analysis includes a pangenome and core genome investigation, complemented with average nucleotide identity (ANI) analysis. Examination of genes associated with pathogenicity provides insight into taxonomic associations with clinical symptoms.

## RESULTS

### Genome analyses support existence of distinct *Gardnerella* species.

To determine if the matrix-assisted laser desorption ionization–time of flight mass spectrometry (MALDI-TOF MS) assignments are indicative of genetic differences, we purified and sequenced the genomes of 10 clinical isolates (6 identified by MALDI-TOF MS as *G. vaginalis* and 4 identified by MALDI-TOF MS as *Gardnerella* species) (see Materials and Methods). These isolates were collected from 10 different women with overactive bladder (OAB) symptoms (*n* = 7), stress urinary incontinence (SUI) (*n* = 1), and diabetes (*n* = 1) and from a kidney stone (*n* = 1) (see Table S1 in the supplemental material). Statistics regarding these 10 new genomes can be found in Table S1. We then compared the 10 new genome assemblies to 103 publicly available *Gardnerella* assemblies for a total of 113 genomes (Table S2). Of the 103 publicly available *Gardnerella* assemblies, 19 were published subsequent to the work of Vaneechoutte et al. (13) and 4 of these strains were deposited as *Gardnerella* species; these were omitted from the ANI analysis of Vaneechoutte et al. (13). These 23 genomes, in addition to the 10 new genomes produced for this study, have not been previously examined. The 10 new genomes presented here, as well as 22 of the 103 publicly available assemblies included in this analysis, come from our own collection. These 32, plus 1 not from our collection, are the only sequenced urinary isolates for the genus; most isolates come from vaginal samples (*n* = 78) (Table S2).

10.1128/mSphere.00154-21.2 TABLE S1 Genome assembly statistics and patient symptom status for new urinary *Gardnerella* isolates. Download Table S1, PDF file, 0.1 MB. Copyright © 2021 Putonti et al. 2021 Putonti et al. https://creativecommons.org/licenses/by/4.0/ This content is distributed under the terms of the Creative Commons Attribution 4.0 International license.

10.1128/mSphere.00154-21.3 TABLE S2 Genomes included in the comparative study of the *Gardnerella* species. Download Table S2, PDF file, 0.1 MB. Copyright © 2021 Putonti et al. 2021 Putonti et al. https://creativecommons.org/licenses/by/4.0/ This content is distributed under the terms of the Creative Commons Attribution 4.0 International license.

**FIG 1:**
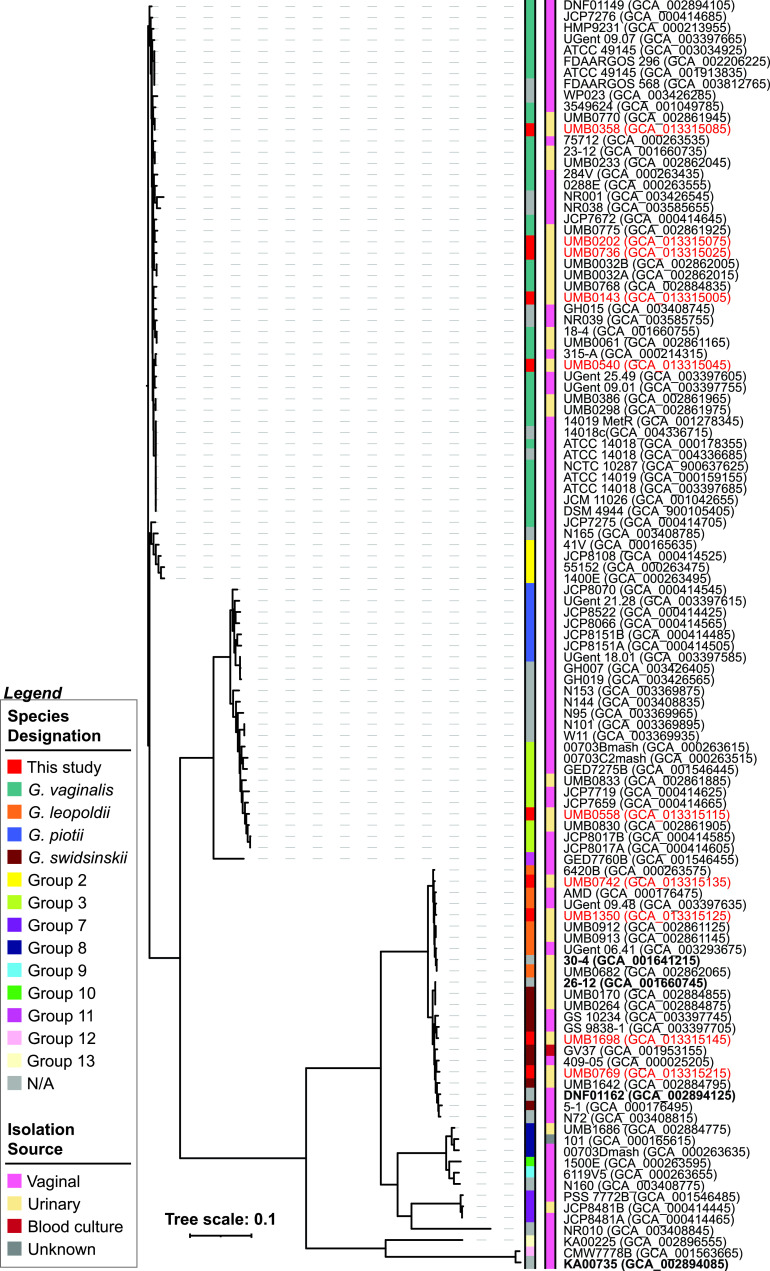
Core genome phylogeny for *Gardnerella* strains. Accession numbers are listed in parentheses. The color bar indicates the genomic species/group per the study by Vaneechoutte et al. (13) or N/A (gray; not included in the study by Vaneechoutte et al. [13]) or new genomes produced in this study (red). Strain names and accession numbers produced as part of this study are listed in red. Strains deposited as “*Gardnerella* species” are listed in bold. Tree scale refers to evolutionary distance based upon the FastTree’s approximate maximum-likelihood method.

The pangenome of the 113 *Gardnerella* assemblies includes 4,542 unique genes, with 1,399 of these genes unique to a single strain assembly. The core genome includes 138 single-copy genes present in all 113 assemblies. The functionalities of these genes were assessed via blastp queries to the complete nr database (Table S3). All of these sequences returned significant hits to annotated *Gardnerella* sequences as expected, and many also exhibited significant homologies to annotated protein records from other species within the family *Bifidobacteriaceae*. The majority (87%) of these core sequences exhibited homology to annotated functions. Using these core gene sequences, we derived a phylogenomic tree (Fig. 1). The clade structure of this tree mirrored that of prior work (13, 14). The same 13 groups as identified by Vaneechoutte et al. (13) were identified in this study. However, our phylogenomic tree also provided insight into classification of new strains, including those published after (or omitted) from the earlier work of Vaneechouttee et al. (13) (Fig. 1, gray) and the 10 new genomes published as part of this study (Fig. 1, red).

10.1128/mSphere.00154-21.4 TABLE S3 *Gardnerella* core gene functionality. Download Table S3, PDF file, 0.1 MB. Copyright © 2021 Putonti et al. 2021 Putonti et al. https://creativecommons.org/licenses/by/4.0/ This content is distributed under the terms of the Creative Commons Attribution 4.0 International license.

**FIG 2:**
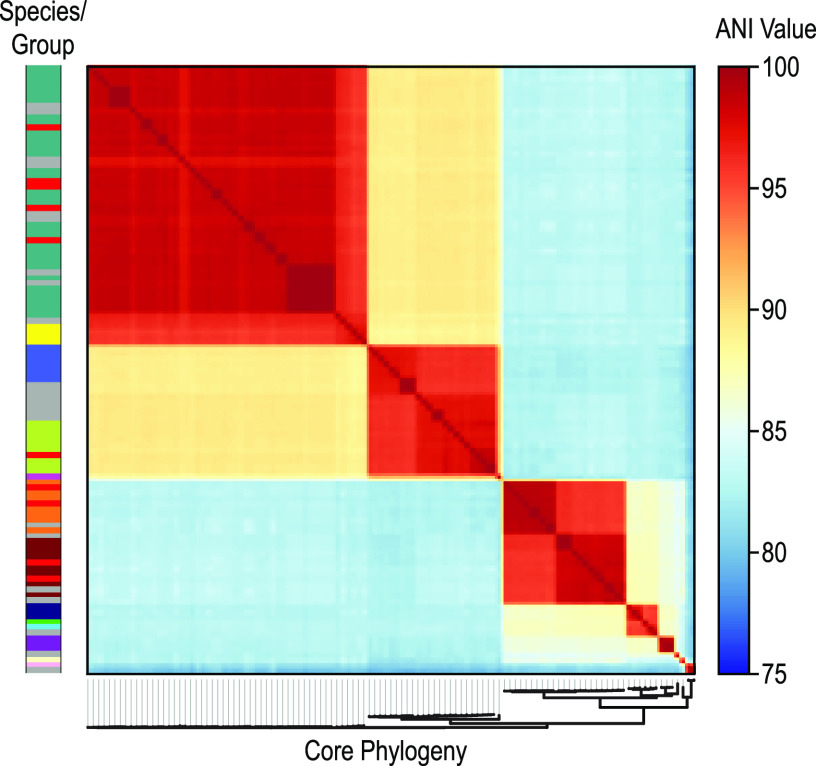
ANI analysis of 113 *Gardnerella* strains. The core phylogeny is shown at the bottom, and the species/group is shown on the left bar (same order and color key as Fig. 1).

Next, we conducted ANI analysis for the 113 genomes (Fig. 2). The ANI analysis also provides insight into the classification of the previously unanalyzed genomes (Fig. 1, gray) and the newly sequenced genomes (Fig. 1, red). The combined phylogenomic and ANI analyses identify a new group: group 14, which is represented by the single member, NR010 (GCA_003408845). While the core phylogeny shows that this strain is most closely related to the group 7 strains, the ANI analysis shows that it is distinctly different (Fig. S1). ANI comparisons within group 7 strains range between 98.87 and 99.96%. In contrast, NR010 versus group 7 strain ANI values range between 85.20 and 85.33%; this is on par with ANI values between groups. Thus, we designate *Gardnerella* strain NR010, isolated from the vaginal mucus from a woman in Kenya (11), as the sole representative of group 14.

10.1128/mSphere.00154-21.1 FIG S1 Core genome phylogeny and ANI comparison for new proposed *Gardnerella* group (gray) and group 7 strains (purple). Download FIG S1, EPS file, 1.8 MB. Copyright © 2021 Putonti et al. 2021 Putonti et al. https://creativecommons.org/licenses/by/4.0/ This content is distributed under the terms of the Creative Commons Attribution 4.0 International license.

### Comparison of pathogenicity genes between the *Gardnerella* species/groups.

Previous work has suggested that mucin degradation, sialidase activity, and vaginolysin activity contribute to *Gardnerella* pathogenicity. The mucin degradation pathway consists of 6 enzymes (Table 1) and includes sialidase activity. Only *G. vaginalis* (*n* = 43/47) and group 2 (*n* = 5/5) strains contained genes that encode the complete mucin degradation pathway; all other species or groups were missing part or all of the pathway (Table 1; Table S4). The genes that encode sialidase A and *O*-sialoglycoprotein endopeptidase were present in *G. vaginalis*, *G. piotii*, and group 2, group 3, and group 11 strains (Table 1). Groups 7 (*n* = 3), 12 (*n* = 2), and 13 (*n* = 1) are omitted from Table 1, as none of the genomes contained recognizable homologs of these 6 gene sequences.

**TABLE 1:** Presence of mucin degradation genes

Species or group	*n*	No. of strains with indicated gene					
		Sialidase A	*O*-Sialoglycoprotein endopeptidase	Beta-galactosidase	Alpha-l-fucosidase	M22 family glycoprotease	Alpha-mannosidase
*G. leopoldii*	10					3	
*G. piotii*	9	5	7			7	7
*G. swidsinskii*	13					1	7
*G. vaginalis*	47	43	44	44	44	44	44
Group 2	5	5	5	5	5	5	5
Group 3	15	15	15			15	15
Group 8	3	3					
Group 9	2	2					
Group 10	1	1					
Group 11	1	1	1			1	1
Group 14	1	1					

10.1128/mSphere.00154-21.5 TABLE S4 Presence of mucin degradation and vaginolysin genes in 113 *Gardnerella* genomes. Download Table S4, PDF file, 0.2 MB. Copyright © 2021 Putonti et al. 2021 Putonti et al. https://creativecommons.org/licenses/by/4.0/ This content is distributed under the terms of the Creative Commons Attribution 4.0 International license.

**FIG 3:**
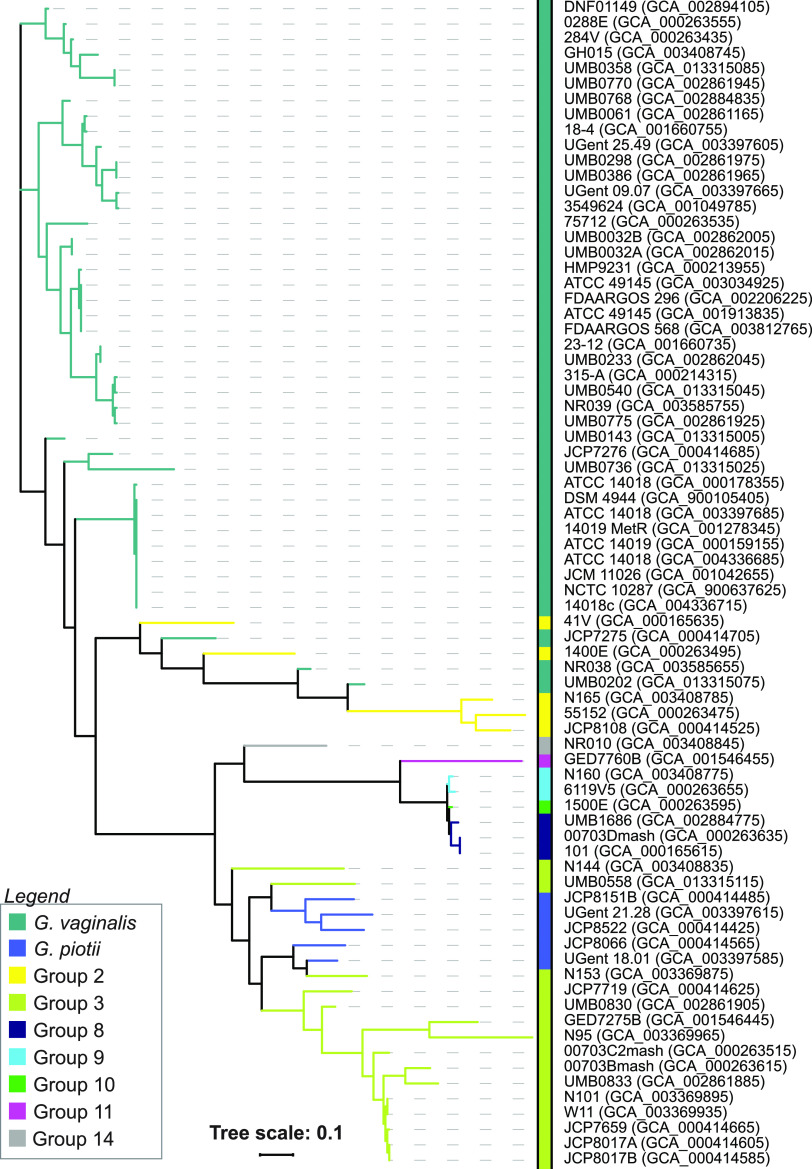
Phylogenetic tree of the sialidase A coding sequences from the *Gardnerella* assemblies. Tree scale refers to evolutionary distance based upon the FastTree’s approximate maximum-likelihood method.

Previously, it was suggested that the presence of the sialidase A gene can be associated with *G. vaginalis* virulence in the vagina (40), and increased sialidase activity within the vaginal fluid of BV^+^ individuals has been observed (41). Genome sequences from 9 of the 14 groups include this gene. A phylogenetic tree of all detected sialidase genes is shown in Fig. 3. While genes from *G. vaginalis* and group 2 were highly related, there remained distinct differences within the *G. vaginalis* group, with one clade exhibiting greater similarity to group 2 sequences than to the other *G. vaginalis* strains. The remaining 7 groups exhibited greater similarity to each other than to the *G. vaginalis* and *G. vaginalis*/group 2 clades.

**FIG 4:**
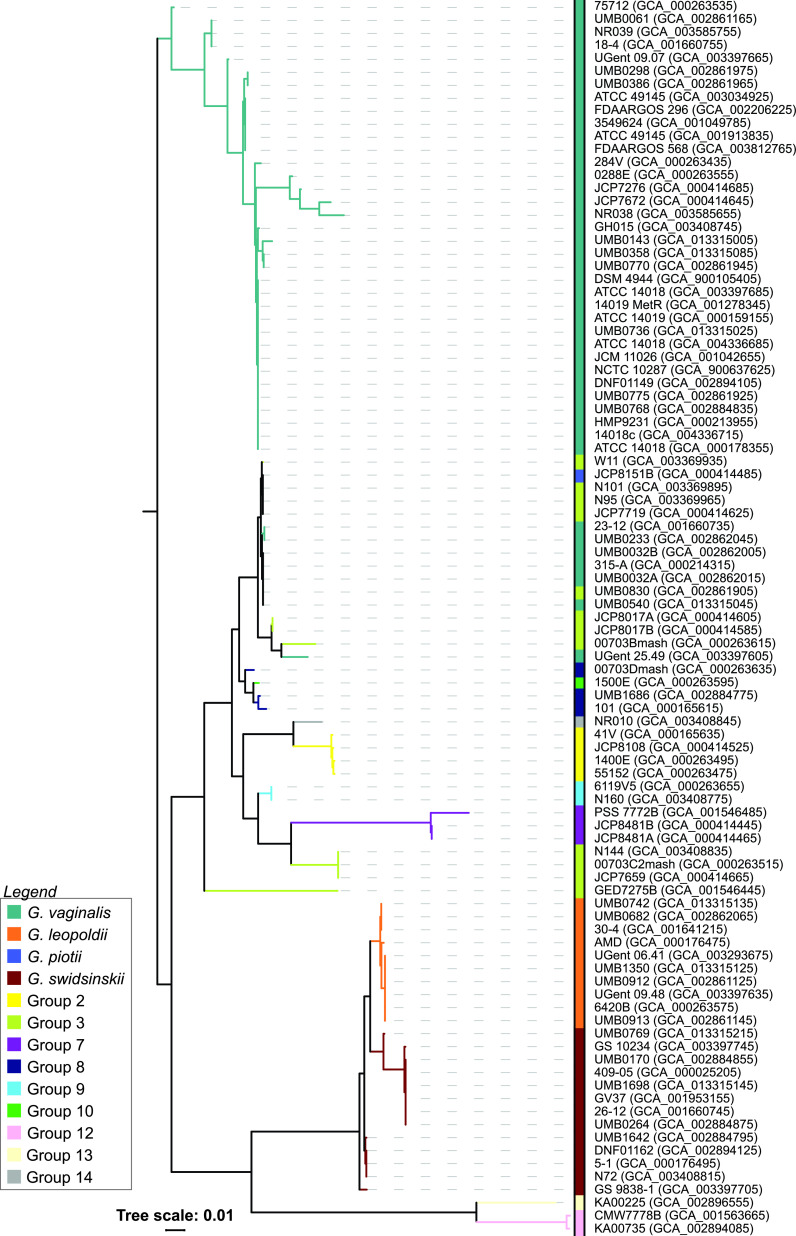
Phylogenetic tree of the vaginolysin coding sequences from the *Gardnerella* assemblies. Tree scale refers to evolutionary distance based upon the FastTree’s approximate maximum-likelihood method.

Vaginolysin (encoded by *vly*) is a cholesterol-dependent cytolysin (CDC) (42). It binds to cholesterol receptors on the surface of vaginal epithelial cells, inducing lysis. It is even hypothesized that the cholesterol level in African American women is what predisposes them to BV (43). Of the 113 genomes examined, the *vly* coding region was detected in 95 genomes (Table S4). Only the single group 11 strain’s genome assembly did not contain *vly*; further sequencing of isolates belonging to this group will provide insight into whether this is characteristic for the group. All other species and groups included strains containing *vly*. As shown in Fig. 4, the *vly* sequence is not always congruent with the core genome phylogeny. Species/groups are not monophyletic in this tree. For instance, group 3 strains (lime green) clade with both *G. vaginalis* and group 7 strains.

### *Gardnerella* species/groups and associations with urinary symptoms.

MALDI-TOF MS analysis separated members the genus *Gardnerella* into two categories: Gardnerella vaginalis and *Gardnerella* species. Isolates of both types were found in catheterized samples from women with and without OAB, and neither *G. vaginalis* nor *Gardnerella* species are associated with either continence or OAB with statistical significance (χ^2^; *P* = 0.161) (Table 2). Thirteen of the 32 sequenced urinary isolates from our collection were identified as *Gardnerella* species via MALDI-TOF MS (Table S5). Based upon our genome analyses, these strains are representative of *G. swidsinskii* (*n* = 6), *G. leopoldii* (*n* = 6), or group 8 (*n* = 1). The remaining 19 sequenced urinary isolates from our collection were identified as *G. vaginalis* via MALDI-TOF MS; genomic analysis determined that 3 were in fact group 3 strains, while the others were *G. vaginalis* strains (Table S5).

**TABLE 2:** Frequency of detection in patient populations^*a*^

MALDI-TOF MS identification	No. (%) of:	
	Continent controls (*n* = 235)	Subjects with OAB (*n* = 304)
*G. vaginalis*	21 (11.51)	35 (8.94)
*Gardnerella* species	7 (0.66)	7 (2.98)
Both *G. vaginalis* and *Gardnerella* species	2 (0.99)	3 (0.85)

a A total of 235 women without lower urinary tract symptoms and 304 women with OAB symptoms were screened for *Gardnerella*. All samples are from catheterized urine.

10.1128/mSphere.00154-21.6 TABLE S5 MALDI-TOF MS predictions for sequenced urinary *Gardnerella* isolates. Download Table S5, PDF file, 0.1 MB. Copyright © 2021 Putonti et al. 2021 Putonti et al. https://creativecommons.org/licenses/by/4.0/ This content is distributed under the terms of the Creative Commons Attribution 4.0 International license.

Given the species/group designations based upon ANI, we investigated the association of the 32 sequenced strains from our collection with urinary symptoms. All 32 of these strains were isolated from urine samples from women and include representatives of *G. vaginalis* (*n* = 16), *G. swidsinskii* (*n* = 6), *G. leopoldii* (*n* = 6), group 3 (*n* = 3), and group 8 (*n* = 1) (Table S5). These 32 strains grouped with strains isolated from the vaginal microbiota (Fig. 1); thus, these species/groups are not unique to the urinary microbiota. As detailed in Table 3, *G. vaginalis*, *G. swidsinskii*, *G. leopoldii*, and group 3 strains were isolated from asymptomatic patients, as well as individuals with urinary symptoms. There was no significant association between *Gardnerella* species/group and urinary symptoms.

**TABLE 3:** Sources of *Gardnerella* urinary isolates from our collection sequenced

Species or group	No. of isolates from indicated subject type						
	Asymptomatic	Asymptomatic (pregnant)	UUI	SUI	OAB	Kidney stone	Diabetes
*G. vaginalis*	3		2	1	9	1	
*G. swidsinskii*	1		1		3		1
*G. leopoldii*		2	1		3		
Group 3		2		1			
Group 8							1

## DISCUSSION

Several previous studies have demonstrated broad genetic diversity within the *Gardnerella* genus (8–11, 13, 14, 44). Whole-genome sequencing of isolates and comparative studies have been pivotal in describing this diversity (8–10, 14, 44). Genome analysis suggests that these species/groups are in fact reproductively isolated (14). We similarly detected broad genetic diversity, identifying a large accessory genome for the genus. Once only a single species represented this genus; now 13 groups, which include three new *Gardnerella* species, have been described (13). Based upon our core genome analysis and ANI comparisons, we suggest a 14th group.

In contrast to recent investigations of the *Gardnerella* core genome (8, 14), we identified a smaller core gene set: 138 genes. Bohr et al. (14) included 608 genes common among 106 assemblies, and Tarracchini et al. (8) included 334 genes common among 72 assemblies. Our smaller core gene set can be contributed to our inclusion of only single-copy-number genes and the threshold for similarity used. Similar to the aforementioned studies, our core was also identified from primarily draft assemblies, i.e., multiple contigs. We did exclude three publicly available genomes from our analysis, as they did not meet the threshold of completeness (see Materials and Methods). Even though a smaller set of genes was considered in this study, our core genome phylogeny is equivalent to these other recent core genome phylogenies. This concurrence further calls for additional formal delineation of species within the genus.

Classification of isolates by the new *Gardnerella* species/groups may help to resolve the debate over the role of *Gardnerella* in BV progression (5). However, this cannot be accomplished via 16S rRNA-based sequence surveys given the >98.5% similarity across the genus (13). One alternative is using another marker gene, e.g., *cpn60* (11, 12) or the sialidase gene, given its association with BV symptoms (4, 41). Despite its putative clinical relevance, our study found that neither the presence (Table 1) nor sequence (Fig. 2) of sialidase can distinguish between *Gardnerella* species/groups. Moreover, a recent study found that presence of the sialidase A gene does not correspond with BV symptoms (18). Similarly, vaginolysin presence or sequence is insufficient (Fig. 3; Table S4). These findings advocate for whole-genome analysis for taxonomic and etiological classification. Recently, Tarracchini et al. (8) showed the viability of this approach; individual *Gardnerella* species/groups were identified in vaginal microbiome data.

Urinary isolates previously identified as *Gardnerella* species by MALDI-TOF MS are members of the newly recognized species *G. swidsinskii* and *G. leopoldii*. While our collection of sequenced *G. vaginalis* strains includes 12 isolates from women with lower urinary symptoms (OAB, *n* = 9; urge urinary incontinence [UUI], *n* = 2; SUI, *n* = 1), it also includes 3 isolates from asymptomatic women. Since these three isolates’ genomes include annotations for virulence factors, including both sialidase and vaginolysin, we cannot speculate that they are commensal strains. As Table 3 shows, no single species/group could be associated with a urinary symptom cohort. Just as others hypothesize that some *Gardnerella* species/groups may contribute to the development (rather than be the sole cause) of BV (see reviews in references 45 and 46), *Gardnerella* species/groups also may contribute to lower urinary tract symptoms. Other factors likely contribute to symptom status, including overall urinary microbiota composition. Further isolation and sequencing of urinary *Gardnerella* strains are needed to determine if this is true or if age, race, or other demographics contribute to symptom status.

In our prior study, we found that many of the species that inhabit the bladder are also found within the vagina, suggesting that the bladder and vaginal microbiotas are interlinked (39). However, in comparison to the vaginal microbiota, only five species/groups have been identified within the lower urinary tract. While some of the 14 species/groups are represented by a single isolate or just a few isolates, we were intrigued to find that *G. piotii* has yet to be identified in the urinary tract; the nine assemblies examined in this study were all collected from the vagina (Table S2). Indeed, current evidence suggests that *G. piotii* is a commensal of the vagina (12). The urinary genomes examined in this study represent only 6 of the 14 species/groups: *G. leopoldii*, *G. swidsinskii*, *G. vaginalis*, group 3, group 7, and group 8. Future efforts to isolate and sequence *Gardnerella* species/groups from the urinary tract will provide insight into our preliminary observations that some species/groups are not (or are infrequent) members of the urinary microbiota.

## MATERIALS AND METHODS

### Culture and identification.

The 10 *Gardnerella* strains sequenced as part of this study were isolated from urine samples collected and processed as part of prior institutional review board (IRB)-approved studies (LU206449, LU207152, LU207102, LUC204195, and LU204133) (24–26, 30, 31, 39, 47, 48). Isolation was performed using the enhanced quantitative urine culture (EQUC) protocol, in which all morphologically distinct colonies are purified and identified using MALDI-TOF mass spectrometry as reported previously (24). MALDI-TOF MS identification of *Gardnerella* isolates was conducted prior to the definition of the 3 new *Gardnerella* species. MALDI-TOF MS peaks have been reassessed subsequent to our analysis such that the four *Gardnerella* species can be distinguished (13). Following isolation, samples were stored down in brucella broth (Hardy Diagnostics, Santa Maria, CA) at −80°C for future use.

### Whole-genome sequencing.

For sequencing, the isolates were struck onto BD BBL CDC anaerobe 5% sheep blood agar and grown anaerobically for 48 h, whereupon the colonies were scraped off the plates, resuspended in phosphate-buffered saline (PBS), and pelleted. Genomic DNA was extracted from pelleted cells using a phenol-chloroform method (49). DNA was prepared and sequenced using the Illumina Hi-Seq platform with library fragment sizes of 200 to 300 bp and a read length of 100 bp at the Wellcome Trust Sanger Institute, as previously described (50). Annotated assemblies were produced using the pipeline described previously (51). Briefly, sequence reads were used to create multiple assemblies using Velvet v1.2 (52) and VelvetOptimiser v2.2.5 (https://github.com/tseemann/VelvetOptimiser). An assembly improvement step was applied to the assembly with the best *N*_50_, and contigs were scaffolded using SSPACE (53) and sequence gaps filled using GapFiller (54). Automated annotation was performed using the NCBI Prokaryotic Genome Annotation Pipeline (PGAP) v4.11 (55) upon submission to GenBank’s Assembly database.

### Core genome and average nucleotide identity analysis.

Publicly available *Gardnerella* genomes were identified from the NCBI (Table S1). Genome assemblies were evaluated for their completeness using CheckM (56). As a result, three publicly available sequences were excluded from evaluation. It is worth noting that the final genome assemblies examined include three different sequencing projects for strain ATCC 14018 and two different sequencing projects for strain ATCC 49145. The pangenome was identified using anvi’o (57). Homologous genes were identified using a Markov clustering (MCL) inflation of 6. Single-copy core genes were identified by anvi’o, and the functionality of these genes was determined via blastp queries (using the core gene amino acid sequence representative of each core gene from the *G. vaginalis* 409-05 annotation) against the nr database. The phylogenomic tree was derived based upon the alignment of the concatenation of the single-copy core gene amino acid sequences using anvi’o (57), FastTree (58), and iTOL (59). ANI was calculated by performing pairwise comparisons of complete genome assemblies using fastANI (60).

### Gene-specific analysis.

Genes that encode mucin degradation, sialidase activity, and vaginolysin were identified for each strain from the PGAP annotations and confirmed by local blastn queries. Gene sequences were aligned with MAFFT v7.388 (61). FastTree (58) and iTOL (59) were used to derive and visualize the phylogenetic relationship for vaginolysin and sialidase.

### Data availability.

Raw reads and genomes have been deposited in NCBI’s SRA and Assembly databases, respectively. Accession numbers for genome assemblies are listed in Table S1 and include GCA_013315005, GCA_013315025, GCA_013315045, GCA_013315075, GCA_013315085, GCA_013315115, GCA_013315125, GCA_013315135, GCA_013315145, and GCA_013315215.

## References

[1] Fredricks DN, Fiedler TL, Marrazzo JM. 2005. Molecular identification of bacteria associated with bacterial vaginosis. N Engl J Med 353:1899–1911. doi:10.1056/NEJMoa043802. 16267321

[2] Nasioudis D, Linhares IM, Ledger WJ, Witkin SS. 2017. Bacterial vaginosis: a critical analysis of current knowledge. BJOG 124:61–69. doi:10.1111/1471-0528.14209. 27396541

[3] Brubaker L, Wolfe AJ. 2017. The female urinary microbiota, urinary health and common urinary disorders. Ann Transl Med 5:34. doi:10.21037/atm.2016.11.62. 28217699 PMC5300856

[4] Janulaitiene M, Paliulyte V, Grinceviciene S, Zakareviciene J, Vladisauskiene A, Marcinkute A, Pleckaityte M. 2017. Prevalence and distribution of Gardnerella vaginalis subgroups in women with and without bacterial vaginosis. BMC Infect Dis 17:394. doi:10.1186/s12879-017-2501-y. 28583109 PMC5460423

[5] Hickey RJ, Forney LJ. 2014. Gardnerella vaginalis does not always cause bacterial vaginosis. J Infect Dis 210:1682–1683. doi:10.1093/infdis/jiu303. 24855684 PMC4334793

[6] Callahan BJ, DiGiulio DB, Goltsman DSA, Sun CL, Costello EK, Jeganathan P, Biggio JR, Wong RJ, Druzin ML, Shaw GM, Stevenson DK, Holmes SP, Relman DA. 2017. Replication and refinement of a vaginal microbial signature of preterm birth in two racially distinct cohorts of US women. Proc Natl Acad Sci U S A 114:9966–9971. doi:10.1073/pnas.1705899114. 28847941 PMC5604014

[7] Pleckaityte M, Janulaitiene M, Lasickiene R, Zvirbliene A. 2012. Genetic and biochemical diversity of *Gardnerella vaginalis* strains isolated from women with bacterial vaginosis. FEMS Immunol Med Microbiol 65:69–77. doi:10.1111/j.1574-695X.2012.00940.x. 22309200

[8] Tarracchini C, Lugli GA, Mancabelli L, Milani C, Turroni F, Ventura M. 2020. Assessing the genomic variability of *Gardnerella vaginalis* through comparative genomic analyses: evolutionary and ecological implications. Appl Environ Microbiol 87:e02188-20. doi:10.1128/AEM.02188-20. 33097505 PMC7755242

[9] Ahmed A, Earl J, Retchless A, Hillier SL, Rabe LK, Cherpes TL, Powell E, Janto B, Eutsey R, Hiller NL, Boissy R, Dahlgren ME, Hall BG, Costerton JW, Post JC, Hu FZ, Ehrlich GD. 2012. Comparative genomic analyses of 17 clinical isolates of Gardnerella vaginalis provide evidence of multiple genetically isolated clades consistent with subspeciation into genovars. J Bacteriol 194:3922–3937. doi:10.1128/JB.00056-12. 22609915 PMC3416530

[10] Cornejo OE, Hickey RJ, Suzuki H, Forney LJ. 2018. Focusing the diversity of Gardnerella vaginalis through the lens of ecotypes. Evol Appl 11:312–324. doi:10.1111/eva.12555. 29632552 PMC5881158

[11] Schellenberg JJ, Paramel Jayaprakash T, Withana Gamage N, Patterson MH, Vaneechoutte M, Hill JE. 2016. Gardnerella vaginalis subgroups defined by cpn60 sequencing and sialidase activity in isolates from Canada, Belgium and Kenya. PLoS One 11:e0146510. doi:10.1371/journal.pone.0146510. 26751374 PMC4709144

[12] Hill JE, Albert AYK, VOGUE Research Group. 2019. Resolution and cooccurrence patterns of Gardnerella leopoldii, G. swidsinskii, G. piotii, and G. vaginalis within the vaginal microbiome. Infect Immun 87:e00532-19. doi:10.1128/IAI.00532-19. 31527125 PMC6867840

[13] Vaneechoutte M, Guschin A, Van Simaey L, Gansemans Y, Van Nieuwerburgh F, Cools P. 2019. Emended description of Gardnerella vaginalis and description of Gardnerella leopoldii sp. nov., Gardnerella piotii sp. nov. and Gardnerella swidsinskii sp. nov., with delineation of 13 genomic species within the genus Gardnerella. Int J Syst Evol Microbiol 69:679–687. doi:10.1099/ijsem.0.003200. 30648938

[14] Bohr LL, Mortimer TD, Pepperell CS. 2020. Lateral gene transfer shapes diversity of Gardnerella spp. Front Cell Infect Microbiol 10:293. doi:10.3389/fcimb.2020.00293. 32656099 PMC7324480

[15] Castro J, Alves P, Sousa C, Cereija T, França Â, Jefferson KK, Cerca N. 2015. Using an in-vitro biofilm model to assess the virulence potential of bacterial vaginosis or non-bacterial vaginosis Gardnerella vaginalis isolates. Sci Rep 5:11640. doi:10.1038/srep11640. 26113465 PMC4481526

[16] Balashov SV, Mordechai E, Adelson ME, Gygax SE. 2014. Identification, quantification and subtyping of Gardnerella vaginalis in noncultured clinical vaginal samples by quantitative PCR. J Med Microbiol 63:162–175. doi:10.1099/jmm.0.066407-0. 24200640

[17] Janulaitiene M, Gegzna V, Baranauskiene L, Bulavaitė A, Simanavicius M, Pleckaityte M. 2018. Phenotypic characterization of Gardnerella vaginalis subgroups suggests differences in their virulence potential. PLoS One 13:e0200625. doi:10.1371/journal.pone.0200625. 30001418 PMC6042761

[18] Shipitsyna E, Krysanova A, Khayrullina G, Shalepo K, Savicheva A, Guschin A, Unemo M. 2019. Quantitation of all four Gardnerella vaginalis clades detects abnormal vaginal microbiota characteristic of bacterial vaginosis more accurately than putative G. vaginalis sialidase A gene count. Mol Diagn Ther 23:139–147. doi:10.1007/s40291-019-00382-5. 30721449 PMC6394432

[19] Swidsinski A, Loening-Baucke V, Swidsinski S, Verstraelen H. 2015. Polymicrobial Gardnerella biofilm resists repeated intravaginal antiseptic treatment in a subset of women with bacterial vaginosis: a preliminary report. Arch Gynecol Obstet 291:605–609. doi:10.1007/s00404-014-3484-1. 25245669

[20] Muzny CA, Blanchard E, Taylor CM, Aaron KJ, Talluri R, Griswold ME, Redden DT, Luo M, Welsh DA, Van Der Pol WJ, Lefkowitz EJ, Martin DH, Schwebke JR. 2018. Identification of key bacteria involved in the induction of incident bacterial vaginosis: a prospective study. J Infect Dis 218:966–978. doi:10.1093/infdis/jiy243. 29718358 PMC6093354

[21] Muzny CA, Taylor CM, Swords WE, Tamhane A, Chattopadhyay D, Cerca N, Schwebke JR. 2019. An updated conceptual model on the pathogenesis of bacterial vaginosis. J Infect Dis 220:1399–1405. doi:10.1093/infdis/jiz342. 31369673 PMC6761952

[22] Gilbert NM, Lewis WG, Li G, Sojka DK, Lubin JB, Lewis AL. 2019. Gardnerella vaginalis and Prevotella bivia trigger distinct and overlapping phenotypes in a mouse model of bacterial vaginosis. J Infect Dis 220:1099–1108. doi:10.1093/infdis/jiy704. 30715405 PMC6736442

[23] Morrill S, Gilbert NM, Lewis AL. 2020. Gardnerella vaginalis as a cause of bacterial vaginosis: appraisal of the evidence from in vivo models. Front Cell Infect Microbiol 10:168. doi:10.3389/fcimb.2020.00168. 32391287 PMC7193744

[24] Hilt EE, McKinley K, Pearce MM, Rosenfeld AB, Zilliox MJ, Mueller ER, Brubaker L, Gai X, Wolfe AJ, Schreckenberger PC. 2014. Urine is not sterile: use of enhanced urine culture techniques to detect resident bacterial flora in the adult female bladder. J Clin Microbiol 52:871–876. doi:10.1128/JCM.02876-13. 24371246 PMC3957746

[25] Pearce MM, Hilt EE, Rosenfeld AB, Zilliox MJ, Thomas-White K, Fok C, Kliethermes S, Schreckenberger PC, Brubaker L, Gai X, Wolfe AJ. 2014. The female urinary microbiome: a comparison of women with and without urgency urinary incontinence. mBio 5:e01283-14. doi:10.1128/mBio.01283-14. 25006228 PMC4161260

[26] Pearce MM, Zilliox MJ, Rosenfeld AB, Thomas-White KJ, Richter HE, Nager CW, Visco AG, Nygaard IE, Barber MD, Schaffer J, Moalli P, Sung VW, Smith AL, Rogers R, Nolen TL, Wallace D, Meikle SF, Gai X, Wolfe AJ, Brubaker L. 2015. The female urinary microbiome in urgency urinary incontinence. Am J Obstet Gynecol 213:347.e1–347.e11. doi:10.1016/j.ajog.2015.07.009. 26210757 PMC4556587

[27] Karstens L, Asquith M, Davin S, Stauffer P, Fair D, Gregory WT, Rosenbaum JT, McWeeney SK, Nardos R. 2016. Does the urinary microbiome play a role in urgency urinary incontinence and its severity? Front Cell Infect Microbiol 6:78. doi:10.3389/fcimb.2016.00078. 27512653 PMC4961701

[28] Malki K, Shapiro JW, Price TK, Hilt EE, Thomas-White K, Sircar T, Rosenfeld AB, Kuffel G, Zilliox MJ, Wolfe AJ, Putonti C. 2016. Genomes of Gardnerella strains reveal an abundance of prophages within the bladder microbiome. PLoS One 11:e0166757. doi:10.1371/journal.pone.0166757. 27861551 PMC5115800

[29] Jacobs KM, Thomas-White KJ, Hilt EE, Wolfe AJ, Waters TP. 2017. Microorganisms identified in the maternal bladder: discovery of the maternal bladder microbiota. AJP Rep 7:e188–e196. doi:10.1055/s-0037-1606860. 28970961 PMC5621969

[30] Price TK, Hilt EE, Thomas-White K, Mueller ER, Wolfe AJ, Brubaker L. 2020. The urobiome of continent adult women: a cross-sectional study. BJOG 127:193–201. doi:10.1111/1471-0528.15920. 31469215 PMC7197444

[31] Price TK, Lin H, Gao X, Thomas-White KJ, Hilt EE, Mueller ER, Wolfe AJ, Dong Q, Brubaker L. 2020. Bladder bacterial diversity differs in continent and incontinent women: a cross-sectional study. Am J Obstet Gynecol 223:729.e1–729.e10. doi:10.1016/j.ajog.2020.04.033. 32380174 PMC7609606

[32] Kramer H, Kuffel G, Thomas-White K, Wolfe AJ, Vellanki K, Leehey DJ, Bansal VK, Brubaker L, Flanigan R, Koval J, Wadhwa A, Zilliox MJ. 2018. Diversity of the midstream urine microbiome in adults with chronic kidney disease. Int Urol Nephrol 50:1123–1130. doi:10.1007/s11255-018-1860-7. 29651696 PMC5986845

[33] Siddiqui H, Lagesen K, Nederbragt AJ, Jeansson SL, Jakobsen KS. 2012. Alterations of microbiota in urine from women with interstitial cystitis. BMC Microbiol 12:205. doi:10.1186/1471-2180-12-205. 22974186 PMC3538702

[34] Jacobs KM, Price TK, Thomas-White K, Halverson T, Davies A, Myers DL, Wolfe AJ. 6 4 2020. Cultivable bacteria in urine of women with interstitial cystitis: (not) what we expected. Female Pelvic Med Reconstr Surg doi:10.1097/SPV.0000000000000854. PMC8088819 32265402

[35] Gottschick C, Deng Z-L, Vital M, Masur C, Abels C, Pieper DH, Wagner-Döbler I. 2017. The urinary microbiota of men and women and its changes in women during bacterial vaginosis and antibiotic treatment. Microbiome 5:99. doi:10.1186/s40168-017-0305-3. 28807017 PMC5554977

[36] Castro J, Machado D, Cerca N. 2016. Escherichia coli and Enterococcus faecalis are able to incorporate and enhance a pre-formed Gardnerella vaginalis biofilm. Pathog Dis 74:ftw007. doi:10.1093/femspd/ftw007. 26782142

[37] Castro J, Machado D, Cerca N. 2019. Unveiling the role of Gardnerella vaginalis in polymicrobial bacterial vaginosis biofilms: the impact of other vaginal pathogens living as neighbors. ISME J 13:1306–1317. doi:10.1038/s41396-018-0337-0. 30670827 PMC6474217

[38] Chen YB, Hochstedler B, Pham TT, Alvarez MA, Mueller ER, Wolfe AJ. 2020. The urethral microbiota: a missing link in the female urinary microbiota. J Urol 204:303–309. doi:10.1097/JU.0000000000000910. 32118507

[39] Thomas-White K, Forster SC, Kumar N, Van Kuiken M, Putonti C, Stares MD, Hilt EE, Price TK, Wolfe AJ, Lawley TD. 2018. Culturing of female bladder bacteria reveals an interconnected urogenital microbiota. Nat Commun 9:1557. doi:10.1038/s41467-018-03968-5. 29674608 PMC5908796

[40] Hardy L, Jespers V, Van den Bulck M, Buyze J, Mwambarangwe L, Musengamana V, Vaneechoutte M, Crucitti T. 2017. The presence of the putative Gardnerella vaginalis sialidase A gene in vaginal specimens is associated with bacterial vaginosis biofilm. PLoS One 12:e0172522. doi:10.1371/journal.pone.0172522. 28241058 PMC5328246

[41] Briselden AM, Moncla BJ, Stevens CE, Hillier SL. 1992. Sialidases (neuraminidases) in bacterial vaginosis and bacterial vaginosis-associated microflora. J Clin Microbiol 30:663–666. doi:10.1128/JCM.30.3.663-666.1992. 1551983 PMC265128

[42] Hotze EM, Tweten RK. 2012. Membrane assembly of the cholesterol-dependent cytolysin pore complex. Biochim Biophys Acta 1818:1028–1038. doi:10.1016/j.bbamem.2011.07.036. 21835159 PMC3243806

[43] Abdelmaksoud AA, Girerd PH, Garcia EM, Brooks JP, Leftwich LM, Sheth NU, Bradley SP, Serrano MG, Fettweis JM, Huang B, Strauss JF, Buck GA, Jefferson KK. 2017. Association between statin use, the vaginal microbiome, and Gardnerella vaginalis vaginolysin-mediated cytotoxicity. PLoS One 12:e0183765. doi:10.1371/journal.pone.0183765. 28846702 PMC5573284

[44] Yeoman CJ, Yildirim S, Thomas SM, Durkin AS, Torralba M, Sutton G, Buhay CJ, Ding Y, Dugan-Rocha SP, Muzny DM, Qin X, Gibbs RA, Leigh SR, Stumpf R, White BA, Highlander SK, Nelson KE, Wilson BA. 2010. Comparative genomics of Gardnerella vaginalis strains reveals substantial differences in metabolic and virulence potential. PLoS One 5:e12411. doi:10.1371/journal.pone.0012411. 20865041 PMC2928729

[45] Hardy L, Cerca N, Jespers V, Vaneechoutte M, Crucitti T. 2017. Bacterial biofilms in the vagina. Res Microbiol 168:865–874. doi:10.1016/j.resmic.2017.02.001. 28232119

[46] Castro J, Jefferson KK, Cerca N. 2020. Genetic heterogeneity and taxonomic diversity among Gardnerella species. Trends Microbiol 28:202–211. doi:10.1016/j.tim.2019.10.002. 31699644

[47] Thomas-White KJ, Hilt EE, Fok C, Pearce MM, Mueller ER, Kliethermes S, Jacobs K, Zilliox MJ, Brincat C, Price TK, Kuffel G, Schreckenberger P, Gai X, Brubaker L, Wolfe AJ. 2016. Incontinence medication response relates to the female urinary microbiota. Int Urogynecol J 27:723–733. doi:10.1007/s00192-015-2847-x. 26423260 PMC5119460

[48] Thomas-White K, Taege S, Limeira R, Brincat C, Joyce C, Hilt EE, Mac-Daniel L, Radek KA, Brubaker L, Mueller ER, Wolfe AJ. 2020. Vaginal estrogen therapy is associated with increased Lactobacillus in the urine of postmenopausal women with overactive bladder symptoms. Am J Obstet Gynecol 223:727.e1–727.e11. doi:10.1016/j.ajog.2020.08.006. 32791124 PMC7609597

[49] Green MR, Sambrook J. 2012. Molecular cloning: a laboratory manual, 4th ed. Cold Spring Harbor Laboratory Press, Cold Spring Harbor, NY.

[50] Harris SR, Feil EJ, Holden MTG, Quail MA, Nickerson EK, Chantratita N, Gardete S, Tavares A, Day N, Lindsay JA, Edgeworth JD, de Lencastre H, Parkhill J, Peacock SJ, Bentley SD. 2010. Evolution of MRSA during hospital transmission and intercontinental spread. Science 327:469–474. doi:10.1126/science.1182395. 20093474 PMC2821690

[51] Page AJ, De Silva N, Hunt M, Quail MA, Parkhill J, Harris SR, Otto TD, Keane JA. 2016. Robust high-throughput prokaryote de novo assembly and improvement pipeline for Illumina data. Microb Genom 2:e000083. doi:10.1099/mgen.0.000083. 28348874 PMC5320598

[52] Zerbino DR, Birney E. 2008. Velvet: algorithms for de novo short read assembly using de Bruijn graphs. Genome Res 18:821–829. doi:10.1101/gr.074492.107. 18349386 PMC2336801

[53] Boetzer M, Henkel CV, Jansen HJ, Butler D, Pirovano W. 2011. Scaffolding pre-assembled contigs using SSPACE. Bioinformatics 27:578–579. doi:10.1093/bioinformatics/btq683. 21149342

[54] Boetzer M, Pirovano W. 2012. Toward almost closed genomes with GapFiller. Genome Biol 13:R56. doi:10.1186/gb-2012-13-6-r56. 22731987 PMC3446322

[55] Tatusova T, DiCuccio M, Badretdin A, Chetvernin V, Nawrocki EP, Zaslavsky L, Lomsadze A, Pruitt KD, Borodovsky M, Ostell J. 2016. NCBI prokaryotic genome annotation pipeline. Nucleic Acids Res 44:6614–6624. doi:10.1093/nar/gkw569. 27342282 PMC5001611

[56] Parks DH, Imelfort M, Skennerton CT, Hugenholtz P, Tyson GW. 2015. CheckM: assessing the quality of microbial genomes recovered from isolates, single cells, and metagenomes. Genome Res 25:1043–1055. doi:10.1101/gr.186072.114. 25977477 PMC4484387

[57] Eren AM, Esen ÖC, Quince C, Vineis JH, Morrison HG, Sogin ML, Delmont TO. 2015. Anvi’o: an advanced analysis and visualization platform for ’omics data. PeerJ 3:e1319. doi:10.7717/peerj.1319. 26500826 PMC4614810

[58] Price MN, Dehal PS, Arkin AP. 2010. FastTree 2—approximately maximum-likelihood trees for large alignments. PLoS One 5:e9490. doi:10.1371/journal.pone.0009490. 20224823 PMC2835736

[59] Letunic I, Bork P. 2019. Interactive Tree Of Life (iTOL) v4: recent updates and new developments. Nucleic Acids Res 47:W256–W259. doi:10.1093/nar/gkz239. 30931475 PMC6602468

[60] Jain C, Rodriguez-R LM, Phillippy AM, Konstantinidis KT, Aluru S. 2018. High throughput ANI analysis of 90K prokaryotic genomes reveals clear species boundaries. Nat Commun 9:5114. doi:10.1038/s41467-018-07641-9. 30504855 PMC6269478

[61] Katoh K, Standley DM. 2013. MAFFT multiple sequence alignment software version 7: improvements in performance and usability. Mol Biol Evol 30:772–780. doi:10.1093/molbev/mst010. 23329690 PMC3603318

