# Supplementary material for: Genome Investigation of Urinary Gardnerella Strains and Their Relationship to Isolates of the Vaginal Microbiota
Source: mSphere. 2021 May 12;6(3):e00154-21. doi: 10.1128/mSphere.00154-21 (PMC8125048; doi:10.1128/mSphere.00154-21)
Supplement: TABLE S1 [file mSphere.00154-21-st001.pdf]

Assembly Accession #	Strain Designation	# Contigs	Length (bp)	N50 (bp)	Coverage (x)	# Protein Coding Genes	Patient Symptom Status*
GCA_013315005	UMB0143	12	1,647,237	520,360	200	1,207	OAB
GCA_013315025	UMB0736	9	1,729,426	733,551	533	1,297	Kidney Stone
GCA_013315045	UMB0540	11	1,640,183	334,314	463	1,220	OAB
GCA_013315075	UMB0202	8	1,655,000	548,912	200	1,224	OAB
GCA_013315085	UMB0358	10	1,696,104	331,191	401	1,273	OAB
GCA_013315115	UMB0558	14	1,556,973	349,838	555	1,190	SUI
GCA_013315125	UMB1350	15	1,502,575	272,788	78	1,115	OAB
GCA_013315135	UMB0742	1	1,472,679	1,472,679	200	1,091	OAB
GCA_013315145	UMB1698	6	1,586,301	1,227,954	420	1,192	Diabetes
GCA_013315215	UMB0769	5	1,552,587	1,445,396	481	1,157	OAB

Symptom status abbreviations: OAB – overactive bladder; SUI – stress urinary incontinence
