# Supplementary material for: Genome Investigation of Urinary Gardnerella Strains and Their Relationship to Isolates of the Vaginal Microbiota
Source: mSphere. 2021 May 12;6(3):e00154-21. doi: 10.1128/mSphere.00154-21 (PMC8125048; doi:10.1128/mSphere.00154-21)
Supplement: TABLE S2 [file mSphere.00154-21-st002.pdf]

**Assembly Accession #**	**Strain Designation**	**Species designation per Vaneechoutte *et al*. (2019)**	**Isolation Source**
GCA_000025205	409-05	*G. swidsinskii*	vagina
GCA_000159155	ATCC 14019	*G. vaginalis*	vagina
GCA_000165615	101	Group 8	unknown
GCA_000165635	41V	Group 2	vagina
GCA_000176475	AMD	*G. leopoldii*	vagina
GCA_000176495	5-1	*G. swidsinskii*	vagina
GCA_000178355	ATCC 14018	*G. vaginalis*	vagina
GCA_000213955	HMP9231	*G. vaginalis*	vagina
GCA_000214315	315-A	*G. vaginalis*	vagina
GCA_000263435	284V	*G. vaginalis*	vagina
GCA_000263475	55152	Group 2	vagina
GCA_000263495	1400E	Group 2	vagina
GCA_000263515	00703C2mash	Group 3	vagina
GCA_000263535	75712	*G. vaginalis*	vagina
GCA_000263555	0288E	*G. vaginalis*	vagina
GCA_000263575	6420B	*G. leopoldii*	vagina
GCA_000263595	1500E	Group 10	vagina
GCA_000263615	00703Bmash	Group 3	vagina
GCA_000263635	00703Dmash	Group 8	vagina
GCA_000263655	6119V5	Group 9	vagina
GCA_000414425	JCP8522	*G. piotii*	vagina
GCA_000414445	JCP8481B	Group 7	vagina
GCA_000414465	JCP8481A	Group 7	vagina
GCA_000414485	JCP8151B	*G. piotii*	vagina
GCA_000414505	JCP8151A	*G. piotii*	vagina
GCA_000414525	JCP8108	Group 2	vagina
GCA_000414545	JCP8070	*G. piotii*	vagina
GCA_000414565	JCP8066	*G. piotii*	vagina
GCA_000414585	JCP8017B	Group 3	vagina
GCA_000414605	JCP8017A	Group 3	vagina
GCA_000414625	JCP7719	Group 3	vagina
GCA_000414645	JCP7672	*G. vaginalis*	vagina
GCA_000414665	JCP7659	Group 3	vagina
GCA_000414685	JCP7276	*G. vaginalis*	vagina
GCA_000414705	JCP7275	*G. vaginalis*	vagina
GCA_001042655	JCM 11026	*G. vaginalis*	vagina
GCA_001049785	3549624	*G. vaginalis*	vaginal swab

**Assembly Accession #**	**Strain Designation**	**Species designation per Vaneechoutte *et al.* (2019)**	**Isolation Source**
GCA_001278345	14019_MetR	*G. vaginalis*	vagina
GCA_001546445	GED7275B	Group 3	vagina
GCA_001546455	GED7760B	Group 11	vagina
GCA_001546485	PSS_7772B	Group 7	urine
GCA_001563665	CMW7778B	Group 12	vagina
GCA_001641215	30-4	N/A*	urine
GCA_001660735	23-12	*G. vaginalis*	urine
GCA_001660745	26-12	N/A*	urine
GCA_001660755	18-4	*G. vaginalis*	urine
GCA_001913835	ATCC 49145	*G. vaginalis*	vagina
GCA_001953155	GV37	*G. swidsinskii*	blood culture
GCA_002206225	FDAARGOS_296	*G. vaginalis*	vagina
GCA_002861125	UMB0912	*G. leopoldii*	urine
GCA_002861145	UMB0913	*G. leopoldii*	urine
GCA_002861165	UMB0061	*G. vaginalis*	urine
GCA_002861885	UMB0833	Group 3	urine
GCA_002861905	UMB0830	Group 3	urine
GCA_002861925	UMB0775	*G. vaginalis*	urine
GCA_002861945	UMB0770	*G. vaginalis*	urine
GCA_002861965	UMB0386	*G. vaginalis*	urine
GCA_002861975	UMB0298	*G. vaginalis*	urine
GCA_002862005	UMB0032B	*G. vaginalis*	urine
GCA_002862015	UMB0032A	*G. vaginalis*	urine
GCA_002862045	UMB0233	*G. vaginalis*	urine
GCA_002862065	UMB0682	*G. leopoldii*	urine
GCA_002884775	UMB1686	Group 8	urine
GCA_002884795	UMB1642	*G. swidsinskii*	urine
GCA_002884835	UMB0768	*G. vaginalis*	urine
GCA_002884855	UMB0170	*G. swidsinskii*	urine
GCA_002884875	UMB0264	*G. swidsinskii*	urine
GCA_002894085	KA00735	N/A*	vagina
GCA_002894105	DNF01149	*G. vaginalis*	vagina
GCA_002894125	DNF01162	N/A*	vagina
GCA_002896555	KA00225	Group 13	vagina
GCA_003034925	ATCC 49145	*G. vaginalis*	vagina
GCA_003293675	UGent 06.41	*G. leopoldii*	vagina
GCA_003369875	N153	N/A	vaginal swab

**Assembly Accession #**	**Strain Designation**	**Species designation per Vaneechoutte *et al*. (2019)**	**Isolation Source**
GCA_003369895	N101	N/A	vaginal swab
GCA_003369935	W11	N/A	vaginal swab
GCA_003369965	N95	N/A	vaginal swab
GCA_003397585	UGent 18.01	*G. piotii*	vagina
GCA_003397605	UGent 25.49	*G. vaginalis*	vagina
GCA_003397615	UGent 21.28	*G. piotii*	vagina
GCA_003397635	UGent 09.48	*G. leopoldii*	vagina
GCA_003397665	UGent 09.07	*G. vaginalis*	vagina
GCA_003397685	ATCC 14018	*G. vaginalis*	vagina
GCA_003397705	GS 9838-1	*G. swidsinskii*	vagina
GCA_003397745	GS 10234	*G. swidsinskii*	vagina
GCA_003397755	UGent 09.01	*G. vaginalis*	vagina
GCA_003408745	GH015	N/A	vaginal mucus
GCA_003408775	N160	N/A	vaginal mucus
GCA_003408785	N165	N/A	vaginal mucus
GCA_003408815	N72	N/A	vaginal mucus
GCA_003408835	N144	N/A	vaginal mucus
GCA_003408845	NR010	N/A	vaginal mucus
GCA_003426285	WP023	N/A	vaginal fluid
GCA_003426405	GH007	N/A	vaginal fluid
GCA_003426545	NR001	N/A	vaginal fluid
GCA_003426565	GH019	N/A	vaginal fluid
GCA_003585655	NR038	N/A	vaginal fluid
GCA_003585755	NR039	N/A	vaginal fluid
GCA_003812765	FDAARGOS_568	N/A	vagina
GCA_004336685	ATCC 14018	N/A	vagina
GCA_004336715	14018c	N/A	vagina
GCA_013315005	UMB0143	N/A (This study)	urine
GCA_013315025	UMB0736	N/A (This study)	urine
GCA_013315045	UMB0540	N/A (This study)	urine
GCA_013315075	UMB0202	N/A (This study)	urine
GCA_013315085	UMB0358	N/A (This study)	urine
GCA_013315115	UMB0558	N/A (This study)	urine
GCA_013315125	UMB1350	N/A (This study)	urine
GCA_013315135	UMB0742	N/A (This study)	urine
GCA_013315145	UMB1698	N/A (This study)	urine
GCA_013315215	UMB0769	N/A (This study)	urine

**Assembly Accession #**	**Strain Designation**	**Species designation per Vaneechoutte *et al*. (2019)**	**Isolation Source**
GCA_900105405	DSM 4944	*G. vaginalis*	vagina
GCA_900637625	NCTC 10287	*G. vaginalis*	vagina

* Deposited as "*Gardnerella* species".
