# Supplementary material for: Genome Investigation of Urinary Gardnerella Strains and Their Relationship to Isolates of the Vaginal Microbiota
Source: mSphere. 2021 May 12;6(3):e00154-21. doi: 10.1128/mSphere.00154-21 (PMC8125048; doi:10.1128/mSphere.00154-21)
Supplement: TABLE S3 [file mSphere.00154-21-st003.pdf]

Function	Top BLAST hit Accession #
hypothetical protein	RFT32212.1
carbonate dehydratase	ADB14052.1
ABC transporter, substrate-binding protein	ADB14110.1
50S ribosomal protein L13	RFT33442.1
thymidylate synthase	ADB14246.1
ribosomal protein L25	ADB14521.1
Raf-like protein	ADB14036.1
ATP-dependent Clp protease proteolytic subunit	EIK77590.1
ABC transporter permease	ADB13988.1
ABC-type metal ion transporter permease	RFT35923.1
50S ribosomal protein L1	EIK77337.1
tRNA (guanosine(46)-N7)-methyltransferase TrmB	RIY27266.1
TIGR00730 family protein	ADB14609.1
DNA-binding protein WhiA	RFT35906.1
hypothetical protein	ADB14697.1
preprotein translocase YajC	ADB13529.1
haloacid dehalogenase	RFT36013.1
hypothetical protein	ADB13737.1
hypothetical protein	ADB14243.1
phosphoribosylaminoimidazolesuccinocarboxamide synthase	ADB14023.1
F0F1 ATP synthase subunit epsilon	EIK74127.1
1-acyl-sn-glycerol-3-phosphate acyltransferase	RIY25557.1
ABC transporter permease	ADB14042.1
hypothetical protein	ADB13836.1
30S ribosomal protein S8	MBF9308677.1
NrdH-redoxin	RIY29085.1
thioredoxin-disulfide reductase	ADB13692.1
5-(carboxyamino)imidazole ribonucleotide mutase	RIY25869.1
hypothetical protein	RFT33393.1
thioredoxin	RFT34072.1
50S ribosomal protein L20	MBF9308815.1
ABC-type amino acid transporter permease	ADB13750.1
repressor LexA	ADB14376.1
co-chaperone YbbN	ADB14162.1
shikimate dehydrogenase	ADB13756.1
cation diffusion facilitator family transporter	ADB14390.1

Function	Top BLAST hit Accession #
bifunctional methylenetetrahydrofolate dehydrogenase/methenyltetrahydrofolate cyclohydrolase	RIY25645.1
carbamoyl-phosphate synthase, small subunit	ADB14535.1
VWA domain-containing protein	ADB14560.1
inositol monophosphatase	RFT35920.1
HAD family phosphatase	RFT35959.1
DNA polymerase III subunit epsilon	ADB13689.1
adenylate kinase	EFH71337.1
ribose-5-phosphate isomerase RpiA	MBF9308707.1
50S ribosomal protein L11	EIK77336.1
ABC transporter permease	ADB13655.1
endonuclease III	RIY28958.1
NAD+ synthase	RFT35926.1
class E sortase	RFT32783.1
AbrB family transcriptional regulator	RIY27068.1
recombination protein RecR	RFT32216.1
TlyA family RNA methyltransferase	RIY29853.1
single-stranded binding family protein	ADB14051.1
GtrA family protein	PMC55662.1
hypothetical protein	ADB13706.1
DeoR-type transcriptional regulator	EFH71567.1
anion transporter	NSX41133.1
cell division ATP-binding protein	ADB14178.1
peroxiredoxin	ADB13731.1
binding-protein-dependent transport systems inner membrane component	EIK77850.1
FHA domain-containing protein	RFT29947.1
ribosome-recycling factor	RFT29939.1
sensor histidine kinase	RFT35036.1
orotidine 5'-phosphate decarboxylase	ADB14007.1
hypothetical protein	ADB13909.1
glutamine synthetase	ADB14623.1
orotate phosphoribosyltransferase	ADB14108.1
pantothenate kinase	ADB14294.1
ABC transporter, ATP-binding	ADB14124.1
HAD hydrolase, family IIB	ADB14629.1
crossover junction endodeoxyribonuclease RuvC	RFT35968.1
orotidine 5'-phosphate decarboxylase	EIK77555.1

Function	Top BLAST hit Accession #
16S rRNA methyltransferase	ADB13822.1
peptide deformylase	MBF9308207.1
methionine adenosyltransferase	ADB13593.1
isochorismatase	RIY26576.1
LysR family transcriptional regulator	RFT35958.1
50S ribosomal protein L23	MBF9308665.1
laccase domain-containing protein	ADB14557.1
HAD family phosphatase	ADB13605.1
50S ribosomal protein L16	MBF9308670.1
transcriptional regulator NrdR	ADB13449.1
ABC transporter	ADB13464.1
DUF3052 domain-containing protein	RIY30314.1
succinate dehydrogenase	ADB14081.1
phenylalanine-tRNA synthetase subunit alpha	ADB13604.1
30S ribosomal protein S13	EPI55792.1
transcriptional regulator	APW19323.1
ABC transporter, ATP-binding	ADB13451.1
uracil phosphoribosyltransferase	KLR95983.1
1-acyl-sn-glycerol-3-phosphate acyltransferase	RIY26568.1
triose-phosphate isomerase	RFT35909.1
hypothetical protein	ADB14415.1
DUF1290 domain-containing protein	RFT29945.1
DUF4418 domain-containing protein	RFT34065.1
50S ribosomal protein L4	RIY27076.1
phosphoglycerate mutase	ADB14600.1
phosphate ABC transporter substrate-binding protein PstS	ADB14366.1
metal ABC transporter permease	RFT38396.1
ribosome-associated translation inhibitor RaiA	ADB14694.1
serine-tRNA ligase	ADB14659.1
hypoxanthine phosphoribosyltransferase	ADB14206.1
methionyl-tRNA formyltransferase	ADB14267.1
RNA pseudouridine synthase	ADB13551.1
aldo/keto reductase	ADB14020.1
NlpC/P60 family protein	ADB13793.1
peptide deformylase	RFT34577.1
RNA polymerase-binding protein RbpA	RFT29951.1
dCTP deaminase	ADB13656.1

Function	Top BLAST hit Accession #
Crp/Fnr family transcriptional regulator	RFT34122.1
hypothetical protein	ADB14681.1
3-dehydroquinate dehydratase	RIY27196.1
two-component system response regulator	APW18648.1
16S rRNA (uracil(1498)-N(3))-methyltransferase	RIY25989.1
tRNA (adenosine(37)-N6)-threonylcarbamoyltransferase complex ATPase subunit type 1 TsaE	ADB14606.1
replicative DNA helicase	NSX31669.1
ABC-type antimicrobial peptide transporter permease	ADB13447.1
CDP-diacylglycerol--glycerol-3-phosphate 3-phosphatidyltransferase	EFH27292.1
PHP domain protein	ADB14683.1
50S ribosomal protein L7/L12	RFT32115.1
amino acid ABC transporter substrate-binding protein	ADB14388.1
guanylate kinase	RIY25943.1
fructosamine kinase	ADB14071.1
YigZ family protein	ADB13642.1
cold-shock DNA-binding domain protein	ADB14268.1
signal recognition particle-docking protein FtsY	ADB13859.1
ribonuclease P protein component	RFT32289.1
lipoprotein signal peptidase	EFH71890.1
anchored repeat-type ABC transporter permease subunit	RIY26569.1
DUF881 domain-containing protein	RFT29944.1
16S rRNA (adenine(1518)-N(6)/adenine(1519)-N(6))-dimethyltransferase	RFT32813.1
AsnC family transcriptional regulator	RFT32234.1
50S ribosomal protein L15	RFT33465.1
ribonuclease HII	RIY27168.1
non-canonical purine NTP pyrophosphatase	ADB14240.1
ribosome-binding factor A	ADB14493.1
division/cell wall cluster transcriptional repressor MraZ	RIY26446.1
