# Supplementary material for: Genome Investigation of Urinary Gardnerella Strains and Their Relationship to Isolates of the Vaginal Microbiota
Source: mSphere. 2021 May 12;6(3):e00154-21. doi: 10.1128/mSphere.00154-21 (PMC8125048; doi:10.1128/mSphere.00154-21)
Supplement: TABLE S4 [file mSphere.00154-21-st004.pdf]

Species/Group	Strain	Sialidase A	O-sialoglycoprotein endopeptidase	beta-galactosidase	alpha-L-fructodase	M22-family glycoprotease	alpha-mannosidase	Vaginolysin
G. leopoldii	30-4	-	-	-	-	+	-	+
G. leopoldii	6420B	-	-	-	-	-	-	+
G. leopoldii	AMD	-	-	-	-	+	-	+
G. leopoldii	UGent 06.41	-	-	-	-	-	-	+
G. leopoldii	UGent 09.48	-	-	-	-	-	-	+
G. leopoldii	UMB0682	-	-	-	-	+	-	+
G. leopoldii	UMB0742	-	-	-	-	-	-	+
G. leopoldii	UMB0912	-	-	-	-	-	-	+
G. leopoldii	UMB0913	-	-	-	-	-	-	+
G. leopoldii	UMB1350	-	-	-	-	-	-	+
G. piotii	GH007	-	-	-	-	-	-	-
G. piotii	GH019	-	-	-	-	-	-	-
G. piotii	JCP8066	+	+	-	-	+	+	-
G. piotii	JCP8070	-	+	-	-	+	+	-
G. piotii	JCP8151A	-	+	-	-	+	+	-
G. piotii	JCP8151B	+	+	-	-	+	+	+
G. piotii	JCP8522	+	+	-	-	+	+	-
G. piotii	UGent 18.01	+	+	-	-	+	+	-
G. piotii	UGent 21.28	+	+	-	-	+	+	-
G. swidsinskii	5-1	-	-	-	-	-	-	+
G. swidsinskii	26-12	-	-	-	-	-	-	+
G. swidsinskii	409-05	-	-	-	-	+	+	+
G. swidsinskii	DNF01162	-	-	-	-	-	+	+
G. swidsinskii	GS 10234	-	-	-	-	-	+	+
G. swidsinskii	GS 9838-1	-	-	-	-	-	-	+
G. swidsinskii	GV37	-	-	-	-	-	+	+
G. swidsinskii	N72	-	-	-	-	-	-	+
G. swidsinskii	UMB0170	-	-	-	-	-	-	+
G. swidsinskii	UMB0264	-	-	-	-	-	-	+
G. swidsinskii	UMB0769	-	-	-	-	-	+	+
G. swidsinskii	UMB1642	-	-	-	-	-	+	+

Species/Group	Strain	Sialidase A	O-sialoglycoprotein endopeptidase	beta-galactosidase	alpha-L-fructodase	M22-family glycoprotease	alpha-mannosidase	Vaginolysin
G. swidsinskii	UMB1698	-	-	-	-	-	+	+
G. vaginalis	75712	+	+	+	+	+	+	+
G. vaginalis	3549624	+	+	+	+	+	+	+
G. vaginalis	0288E	+	+	+	+	+	+	+
G. vaginalis	14018c	+	+	+	+	+	+	+
G. vaginalis	14019_MetR	+	+	+	+	+	+	+
G. vaginalis	18-4	+	+	+	+	+	+	+
G. vaginalis	23-12	+	+	+	+	+	+	+
G. vaginalis	284V	+	+	+	+	+	+	+
G. vaginalis	315-A	+	+	+	+	+	+	+
G. vaginalis	ATCC 14018	+	+	+	+	+	+	+
G. vaginalis	ATCC 14018	+	+	+	+	+	+	+
G. vaginalis	ATCC 14018	+	+	+	+	+	+	+
G. vaginalis	ATCC 14019	+	+	+	+	+	+	+
G. vaginalis	ATCC 49145	+	+	+	+	+	+	+
G. vaginalis	ATCC 49145	+	+	+	+	+	+	+
G. vaginalis	DNF01149	+	+	+	+	+	+	+
G. vaginalis	DSM 4944	+	+	+	+	+	+	+
G. vaginalis	FDAARGOS_296	+	+	+	+	+	+	+
G. vaginalis	FDAARGOS_568	+	+	+	+	+	+	+
G. vaginalis	GH015	+	+	+	+	+	+	+
G. vaginalis	HMP9231	+	+	+	+	+	+	+
G. vaginalis	JCM 11026	+	+	+	+	+	+	+
G. vaginalis	JCP7275	+	+	+	+	+	+	-
G. vaginalis	JCP7276	+	+	+	+	+	+	+
G. vaginalis	JCP7672	-	+	+	+	+	+	+
G. vaginalis	NCTC 10287	+	+	+	+	+	+	+
G. vaginalis	NR001	-	-	-	-	-	-	-
G. vaginalis	NR038	+	+	+	+	+	+	+
G. vaginalis	NR039	+	+	+	+	+	+	+
G. vaginalis	UGent 09.01	-	-	-	-	-	-	-

Species/Group	Strain	Sialidase A	O-sialoglycoprotein endopeptidase	beta-galactosidase	alpha-L-fructodase	M22-family glycoprotease	alpha-mannosidase	Vaginolysin
G. vaginalis	UGent 09.07	+	+	+	+	+	+	+
G. vaginalis	UGent 25.49	+	+	+	+	+	+	+
G. vaginalis	UMB0032A	+	+	+	+	+	+	+
G. vaginalis	UMB0032B	+	+	+	+	+	+	+
G. vaginalis	UMB0061	+	+	+	+	+	+	+
G. vaginalis	UMB0143	+	+	+	+	+	+	+
G. vaginalis	UMB0202	+	+	+	+	+	+	-
G. vaginalis	UMB0233	+	+	+	+	+	+	+
G. vaginalis	UMB0298	+	+	+	+	+	+	+
G. vaginalis	UMB0358	+	+	+	+	+	+	+
G. vaginalis	UMB0386	+	+	+	+	+	+	+
G. vaginalis	UMB0540	+	+	+	+	+	+	+
G. vaginalis	UMB0736	+	+	+	+	+	+	+
G. vaginalis	UMB0768	+	+	+	+	+	+	+
G. vaginalis	UMB0770	+	+	+	+	+	+	+
G. vaginalis	UMB0775	+	+	+	+	+	+	+
G. vaginalis	WP023	-	-	-	-	-	-	-
Group 2	55152	+	+	+	+	+	+	+
Group 2	1400E	+	+	+	+	+	+	+
Group 2	41V	+	+	+	+	+	+	+
Group 2	JCP8108	+	+	+	+	+	+	+
Group 2	N165	+	+	+	+	+	+	-
Group 3	00703Bmash	+	+	-	-	+	+	+
Group 3	00703C2mash	+	+	-	-	+	+	+
Group 3	GED7275B	+	+	-	-	+	+	+
Group 3	JCP7659	+	+	-	-	+	+	+
Group 3	JCP7719	+	+	-	-	+	+	+
Group 3	JCP8017A	+	+	-	-	+	+	+
Group 3	JCP8017B	+	+	-	-	+	+	+
Group 3	N101	+	+	-	-	+	+	+
Group 3	N144	+	+	-	-	+	+	+

Species/Group	Strain	Sialidase A	O-sialoglycoprotein endopeptidase	beta-galactosidase	alpha-L-fructodase	M22-family glycoprotease	alpha-mannosidase	Vaginolysin
Group 3	N153	+	+	-	-	+	+	-
Group 3	N95	+	+	-	-	+	+	+
Group 3	UMB0558	+	+	-	-	+	+	-
Group 3	UMB0830	+	+	-	-	+	+	+
Group 3	UMB0833	+	+	-	-	+	+	-
Group 3	W11	+	+	-	-	+	+	+
Group 7	JCP8481A	-	-	-	-	-	-	+
Group 7	JCP8481B	-	-	-	-	-	-	+
Group 7	PSS_7772B	-	-	-	-	-	-	+
Group 8	101	+	-	-	-	-	-	+
Group 8	00703Dmash	+	-	-	-	-	-	+
Group 8	UMB1686	+	-	-	-	-	-	+
Group 9	6119V5	+	-	-	-	-	-	+
Group 9	N160	+	-	-	-	-	-	+
Group 10	1500E	+	-	-	-	-	-	+
Group 11	GED7760B	+	+	-	-	+	+	-
Group 12	CMW7778B	-	-	-	-	-	-	+
Group 12	KA00735	-	-	-	-	-	-	+
Group 13	KA00225	-	-	-	-	-	-	+
Group 14	NR010	+	-	-	-	-	-	+
