# Supplementary material for: Genome Investigation of Urinary Gardnerella Strains and Their Relationship to Isolates of the Vaginal Microbiota
Source: mSphere. 2021 May 12;6(3):e00154-21. doi: 10.1128/mSphere.00154-21 (PMC8125048; doi:10.1128/mSphere.00154-21)
Supplement: TABLE S5 [file mSphere.00154-21-st005.pdf]

**Strain Designation**	**MALDI-TOF Designation**	**Genomic Designation**	**Patient Symptom Status**
18-4	*G. vaginalis*	*G. vaginalis*	UUI
23-12	*G. vaginalis*	*G. vaginalis*	UUI
26-12	*Gardnerella* species	*G. swidsinskii*	UUI
30-4	*Gardnerella* species	*G. leopoldii*	UUI
UMB0032A	*G. vaginalis*	*G. vaginalis*	Asymptomatic
UMB0032B	*G. vaginalis*	*G. vaginalis*	Asymptomatic
UMB0061	*G. vaginalis*	*G. vaginalis*	OAB
UMB0143	*G. vaginalis*	*G. vaginalis*	OAB
UMB0170	*Gardnerella* species	*G. swidsinskii*	OAB
UMB0202	*G. vaginalis*	*G. vaginalis*	OAB
UMB0233	*G. vaginalis*	*G. vaginalis*	OAB
UMB0264	*Gardnerella* species	*G. swidsinskii*	OAB
UMB0298	*G. vaginalis*	*G. vaginalis*	OAB
UMB0358	*G. vaginalis*	*G. vaginalis*	OAB
UMB0386	*G. vaginalis*	*G. vaginalis*	OAB
UMB0540	*G. vaginalis*	*G. vaginalis*	OAB
UMB0558	*G. vaginalis*	Group 3	SUI
UMB0682	*Gardnerella* species	*G. leopoldii*	OAB
UMB0736	*G. vaginalis*	*G. vaginalis*	Kidney Stone
UMB0742	*Gardnerella* species	*G. leopoldii*	OAB
UMB0768	*G. vaginalis*	*G. vaginalis*	Asymptomatic
UMB0769	*Gardnerella* species	*G. swidsinskii*	OAB
UMB0770	*G. vaginalis*	*G. vaginalis*	OAB
UMB0775	*G. vaginalis*	*G. vaginalis*	SUI
UMB0830	*G. vaginalis*	Group 3	Asymptomatic (Pregnant)
UMB0833	*G. vaginalis*	Group 3	Asymptomatic (Pregnant)
UMB0912	*Gardnerella* species	*G. leopoldii*	Asymptomatic (Pregnant)
UMB0913	*Gardnerella* species	*G. leopoldii*	Asymptomatic (Pregnant)
UMB1350	*Gardnerella* species	*G. leopoldii*	OAB
UMB1642	*Gardnerella* species	*G. swidsinskii*	Asymptomatic
UMB1686	*Gardnerella* species	Group 8	Diabetes
UMB1698	*Gardnerella* species	*G. swidsinskii*	Diabetes
